# Identifying the optimal deep learning architecture and parameters for automatic beam aperture definition in 3D radiotherapy

**DOI:** 10.1002/acm2.14131

**Published:** 2023-09-05

**Authors:** Skylar S. Gay, Kelly D. Kisling, Brian M. Anderson, Lifei Zhang, Dong Joo Rhee, Callistus Nguyen, Tucker Netherton, Jinzhong Yang, Kristy Brock, Anuja Jhingran, Hannah Simonds, Ann Klopp, Beth M. Beadle, Laurence E. Court, Carlos E. Cardenas

**Affiliations:** ^1^ Department of Radiation Physics The University of Texas MD Anderson Cancer Center Houston Texas USA; ^2^ University of California San Diego San Diego California USA; ^3^ Department of Imaging Physics The University of Texas MD Anderson Cancer Center Houston Texas USA; ^4^ Department of Radiation Oncology The University of Texas MD Anderson Cancer Center Houston Texas USA; ^5^ University Hospitals Plymouth NHS Trust Plymouth United Kingdom; ^6^ Department of Radiation Oncology Stanford University Palo Alto California USA; ^7^ Department of Radiation Oncology The University of Alabama at Birmingham Birmingham Alabama USA

**Keywords:** automatic segmentation, cervical cancer, digitally reconstructed radiograph, radiotherapy

## Abstract

**Purpose:**

Two‐dimensional radiotherapy is often used to treat cervical cancer in low‐ and middle‐income countries, but treatment planning can be challenging and time‐consuming. Neural networks offer the potential to greatly decrease planning time through automation, but the impact of the wide range of hyperparameters to be set during training on model accuracy has not been exhaustively investigated. In the current study, we evaluated the effect of several convolutional neural network architectures and hyperparameters on 2D radiotherapy treatment field delineation.

**Methods:**

Six commonly used deep learning architectures were trained to delineate four‐field box apertures on digitally reconstructed radiographs for cervical cancer radiotherapy. A comprehensive search of optimal hyperparameters for all models was conducted by varying the initial learning rate, image normalization methods, and (when appropriate) convolutional kernel size, the number of learnable parameters via network depth and the number of feature maps per convolution, and nonlinear activation functions. This yielded over 1700 unique models, which were all trained until performance converged and then tested on a separate dataset.

**Results:**

Of all hyperparameters, the choice of initial learning rate was most consistently significant for improved performance on the test set, with all top‐performing models using learning rates of 0.0001. The optimal image normalization was not consistent across architectures. High overlap (mean Dice similarity coefficient = 0.98) and surface distance agreement (mean surface distance < 2 mm) were achieved between the treatment field apertures for all architectures using the identified best hyperparameters. Overlap Dice similarity coefficient (DSC) and distance metrics (mean surface distance and Hausdorff distance) indicated that DeepLabv3+ and D‐LinkNet architectures were least sensitive to initial hyperparameter selection.

**Conclusion:**

DeepLabv3+ and D‐LinkNet are most robust to initial hyperparameter selection. Learning rate, nonlinear activation function, and kernel size are also important hyperparameters for improving performance.

## 1 INTRODUCTION

Cervical cancer develops in over half a million women every year worldwide. Most new cases occur in low‐ and middle‐income countries (LMICs) where routine cervical cancer screenings are not common clinical practice.^1^, ^2^, ^3^ Therefore, patients with cervical cancer in LMICs usually present with advanced disease. In a recent report, the American Society of Clinical Oncology and the International Atomic and Energy Agency recommended the use of a four‐field box radiotherapy technique as the primary intervention for invasive cervical cancer in LMICs.^4^, ^5^ Following these guidelines, clinicians manually define these treatment fields based on standard bony landmarks, which can be seen on a patient's digitally reconstructed radiographs (DRRs). Although the treatment field definition process can be performed quickly, it could take up to a few days to complete after patients’ computed tomography (CT) images become available, potentially delaying treatment commencement, which has been linked to poorer overall survival outcomes.^6^ In addition, staff shortages and a lack of resources have hindered the availability and access to these treatments in LMICs.^7^

To address these problems, Kisling et al. developed the first fully automated treatment planning tool for external‐beam radiotherapy in locally advanced cervical cancers.^8^ In that study, the authors introduced a deployable treatment planning solution for gross tumor and at‐risk regions in the pelvis. Bony anatomy in the pelvis (pelvic bones, femoral heads, sacrum, and L4 and L5 vertebral bodies) is segmented using a multi‐atlas–based approach,^9^, ^10^, ^11^ projected into each beam's eye view, and used to automatically identify visible bony landmarks and set the beam aperture's borders using user‐defined rules. Although this approach provided clinically acceptable treatment fields for more than 90% of patients, it was a computational bottleneck in the fully automated process and, due to the manual nature of designing and implementing hard‐coded rules; it showed a lack of robustness toward outliers.

Recently, deep convolutional neural networks (DCNNs) have become the state of the art for medical imaging segmentation. In the context of radiotherapy treatment planning, DCNNs have been very promising for the automation of various contouring and planning tasks.^12^, ^13^ Nevertheless, very few studies have focused on the auto‐segmentation of radiotherapy treatment targets^13^, ^14^, ^15^ and, to the best of our knowledge, only our previous studies have thoroughly investigated the use of DCNNs to auto‐segment treatment fields for use in three‐dimensional (3D) conformal radiotherapy.^16^, ^17^ The reliability of these segmentation tasks is particularly important because their predicted outcomes could have important ramifications for tumor control and related toxicities.

In addition, many studies focus on architectural novelty. These compare the performance of a specially‐designed approach with a few general‐purpose architectures and do not report the impact of a wide range of hyperparameter choices on their results. While this is typically done for consistency, this creates a lack of information in the literature of the impact of hyperparameter choices on model performance and may lead future researchers to initialize hyperparameters based upon convention rather than empirical results.

Therefore, the aims of the current study are twofold. Our primary focus is not architectural novelty but rather identifying the combinations of six classes of hyperparameters that led to the best performance in six commonly‐used 2D DCNN autosegmentation models, and to show which architectures are most robust to initial hyperparameter selection. We additionally identified the best‐performing model for automatically defining radiotherapy treatment fields on DRR images. Because many radiotherapy treatment sites also employ DRR‐defined field apertures (e.g., rectum, brain) for treatment planning, we expect the findings of the current study to be translatable to other treatment sites using 3D conformal radiotherapy.

## 2 MATERIALS & METHODS

### 2.1 Patient data and model input generation

**FIGURE 1:**
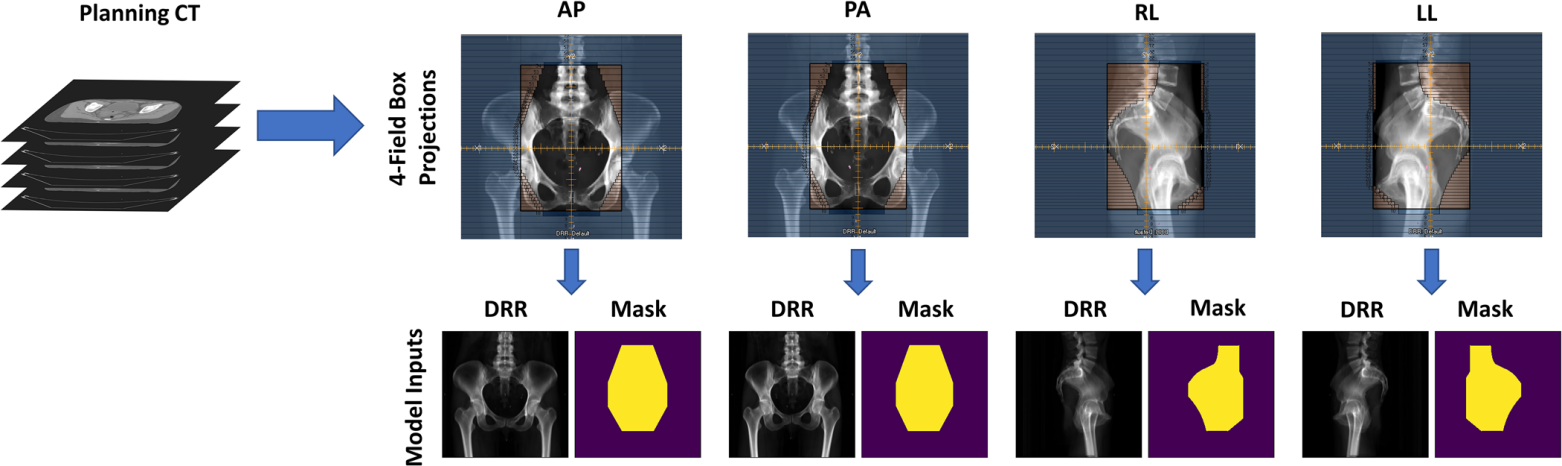
Four‐field conversion process for the 310 digitally reconstructed radiographs (DRRs) used in our study. AP, anterior‐posterior; CT, computed tomography; PA, posterior‐anterior; RL, right‐lateral; LL, left‐lateral.

Simulation CT scans and radiotherapy treatment plans from 310 patients with cervical cancer previously treated at our institution were retrospectively used in this study after institutional review board approval. All patients had physician‐approved four‐field box radiotherapy treatment plans generated by the Radiation Planning Assistant, an automated treatment planning platform.^18^ DRRs were created using the Radiation Planning Assistant^18^, ^19^ and had an isotropic pixel length of 0.68 mm with a resulting matrix size of 512 × 512 (field of view 350 mm). The treatment fields were defined on four orthogonal fields [right‐lateral (RL), left‐lateral (LL), anterior‐posterior (AP), and posterior‐anterior (PA) views] using their respective DRRs. These treatment fields were converted to binary masks using in‐house software (Figure 1) to prepare for segmentation.

The 310 cases used in the study were randomly split into training (*n* = 230), validation (*n* = 25), and final test (*n* = 55) datasets. The validation set was used to evaluate each model's performance during training, and final results were reported for the final test dataset, which was excluded from any training and evaluation done during the training phase. The DRRs were the image inputs to all models, and one‐hot encodings of the treatment field binary masks were the autosegmentation ground truths. All models were trained on three orthogonal fields (AP, PA, and RL) as separate inputs; LL DRRs were excluded as they were simply horizontally mirrored images of the RL treatment fields and therefore do not require autosegmentation to generate.

### 2.2 Training and model parameters investigated

Six commonly used deep learning architectures were evaluated: (1) DeepLabv3+ with Xception backbone,^20^, ^21^ (2) D‐LinkNet,^22^ (3) Res‐U‐Net with residual connections (both element‐wise addition and concatenation), (4) U‐Net^23^ with ReLU activation function, (5) U‐Net with PReLU^24^ activation function, and (6) U‐Net with VGG19^25^ backbone. A variety of parameters were investigated for these architectures (summarized in Table 1); U‐Net and Res‐U‐Net provided the largest combination of possible parameters. All inner convolutions were immediately followed by batch normalization^26^ and a nonlinear activation function (either ReLU or PReLU). All models used softmax as the output activation function and were trained using a batch size of 4 and Dice loss function.^27^ Adam^28^ was chosen as the optimizer for all architectures using the default *α* and *β* values. The impact of training models with different learning rates was explored (learning rate values included 0.01, 0.001, and 0.0001). Network depth was explored for all U‐Net–like architectures; for our analysis, network depth was defined as the number of down sampling steps plus the bottleneck layer of the encoder‐decoder architecture. Depth values explored ranged from 3 to 6 levels. Additionally, kernel sizes of 3×3 and 5×5 were evaluated for these architectures, as well as the initial number of filters (16, 32, 48, and 64) as permitted by graphics processing unit (GPU) memory.

**TABLE 1:** Architectures and architecture‐specific parameters evaluated in our study.

Architecture	Learning rates	Depth	Kernel size	No. of filters	Variations
DeepLabv3+	0.01, 0.001, 0.0001	–	–	–	21
D‐LinkNet	0.01, 0.001, 0.0001	–	–	–	21
Res‐U‐Net (concat)	0.01, 0.001, 0.0001	3, 4, 5, 6	3×3, 5×5	16, 32	336
Res‐U‐Net (add)	0.01, 0.001, 0.0001	3, 4, 5, 6	3×3, 5×5	16, 32	336
U‐Net	0.01, 0.001, 0.0001^a^	3, 4, 5, 6	3×3, 5×5	16, 32, 48, 64	560
U‐Net (PReLU)	0.001, 0.0001^b^	3, 4, 5, 6	3×3, 5×5	16, 32, 48, 64	448
U‐Net (VGG19)	0.01, 0.001, 0.0001	–	–	–	21

^a^ Only 0.001 and 0.0001 learning rate values were evaluated for the 5×5 kernel size.

^b^Only 0.001 and 0.0001 learning rate values were evaluated for this architecture.

The impact of image normalization was also investigated. When DRRs are generated, the DRR's pixel values represent the x‐ray attenuation by voxels encountered along each projection ray from the virtual image source; therefore, absolute image intensities lack physical meaning (unlike Hounsfield units in CT which are related to attenuation coefficients) and image intensity histograms vary widely between patients depending on patient size and other anatomic factors. Three intensity normalization techniques were evaluated in our analysis; these included z‐score normalization (Equation 1), histogram stretching (Equation 2), and L_2_ normalization (Equation 3):

(1) $$I_{new}=\frac{I-\bar{I}}{\sigma_{I}}$$

(2) $$I_{new}=\frac{I-I_{bottom}}{I_{top}-I_{bottom}}\;\left(I_{new,max}-I_{new,min}\right)+I_{bottom}$$

(3) $$I_{new}=\frac{I}{{∥I∥}_{2}}$$

For z‐score normalization, both global and local statistics were evaluated using intensity mean ($\bar{I}$) and standard deviation (σ*_I_*) values, which were calculated using the pixel intensities of all training images or using individual image's pixel intensities, respectively. For histogram stretching, intensity values were normalized such that new intensities were within [0, 1] (i.e., *I_new,max_* = 1 and *I_new,min_* = 0). Four combinations of intensity values were chosen for *I_bottom_* and *I_top_* [global values (*I_bottom_*, *I_top_*) = (0, 45), (0, 90), or (25, 60), and local values (*I_bottom_*, *I_top_*) = (*I_min_*, *I_max_*), where *I_min_* and *I_max_* are the minimum and maximum intensity values, respectively, for an individual DRR image], and all values > *I*_*top*_ were set to have intensity values equal to *I*_*top*_. For L_2_ normalization, we calculated the Euclidean norm (${∥I∥}_{2}=\sqrt{I_{1}^{2}+I_{2}^{2}+\cdots +I_{n}^{2}}\;$) using individual DRR pixel intensities. Following the nomenclature previously established, this was a local statistic, and no attempts were made to normalize on a global scale. Combined, a total of 7 intensity normalization schemes were evaluated.

All models were trained on a 16‐GB Nvidia Tesla V100 GPU with Keras 2.2.2 (TensorFlow 1.11.0 backend).^29^, ^30^ Models were trained to 1500 epochs using early‐stopping regularization based on loss metrics calculated on the validation dataset.

### 2.3 Evaluation metrics

The predicted treatment fields were compared with the clinically defined treatment fields. The Dice similarity coefficient^31^ (DSC), mean surface distance (MSD), and Hausdorff distance (HD) were calculated. These metrics are defined as follows:

(4) $$\mathrm{DSC}\;=\frac{2*\left|A\cap B\right|}{\left|A\right|+\left|B\right|}$$

(5) $$\mathrm{MSD}\;=\frac{1}{2}\;\left({\bar{d}}_{A,B}+{\bar{d}}_{B,A}\right)$$

(6) $$\mathrm{HD}\;=\;\max\left(d_{A,B}d_{B,A}\right)$$

where |*A*| and |*B*| are the number of voxels from contoured volumes *A* and *B*, respectively; |*A*∩*B*| denotes the number of voxels included in the intersection between volumes *A* and *B*; *d_A,B_* is a vector containing all minimum Euclidian surface distances from the surface point from volume *A* to *B*; and ${\bar{d}}_{A,B}$ is the average value in the vector *d*. DSC values range from 0 (no overlap) to 1 (perfect overlap); for both MSD and HD, values closer to zero represent better agreement between two contours’ surfaces. The raw model predictions (no post‐processing was applied) were used to calculate these metrics. Pearson correlation coefficients were calculated to identify trends in quantitative metrics during data analysis.

## 3 RESULTS

In total, 1743 of 2527 potential models were trained to exhaustively evaluate individual architectures, architecture‐specific parameters, learning rates, and image normalization approaches (Table 1). GPU memory limitations prevented the remaining 784 potential models from training; these included the U‐Net–based models that used 5×5 kernel sizes with an initial number of filters greater than 32.

**FIGURE 2:**
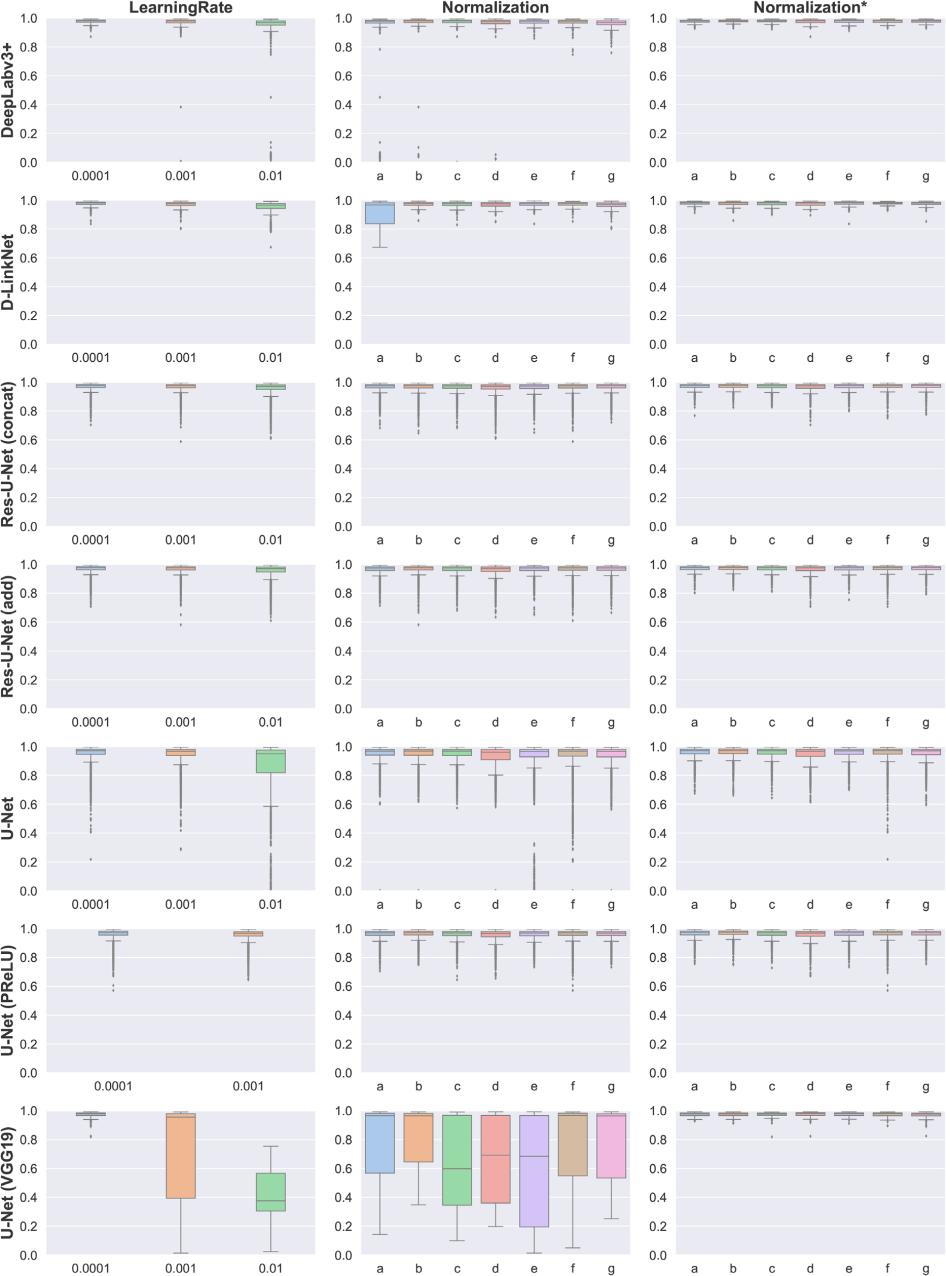
Dice similarity coefficient (DSC) values for each architecture (rows) by learning rate and image intensity normalization scheme (columns). Normalization schemes are abbreviated as follows: a, z‐score normalization with global values (μ = 25 and σ = 30); b, z‐score normalization with individual image values; c, histogram stretching (*I_bottom_* = 25, *I_top_* = 60); d, histogram stretching (*I_bottom_* = 0, *I_top_* = 45); e, histogram stretching (*I_bottom_* = 0, *I_top_* = 90); f, histogram stretching (*I_bottom_* = *I_min_*, *I_top_* = *I_max_*); g, L_2_ normalization. The middle column (Normalization) contains results for models trained with all learning rates, while the right column (Normalization*) includes only results for models trained with a learning rate value of 0.0001.

There was a slight negative correlation between the initial learning rate and overall performance based on DSC values (Pearson correlation coefficient = −0.24; Figure 2) and a slight positive correlation between learning rate and MSD and HD values (Pearson correlation coefficient = 0.14 for MSD and 0.12 for HD), but some architectures were more robust to the investigated learning rates (Figure 2, second row). Model performance was less sensitive to the intensity normalization approaches investigated, and no clearly superior intensity normalization scheme was identified.

The mean DSC, MSD, and HD values for the architectures investigated are summarized in Table 2. On average, the DeepLabV3+ and D‐LinkNet architectures provided the most consistent results during the hyperparameter search; this was most notable in the HD values. After selecting optimal hyperparameters on the test dataset, we noticed no statistical difference between the top models’ performance (Table 3).

**TABLE 2:** Performance of the architectures examined in our study.

Architecture	Mean ± SD across all models			Mean ± SD for the best models		
	DSC	MSD, mm	HD, mm	DSC	MSD, mm	HD, mm
DeepLabv3+	0.958 ± 0.111	3.0 ± 5.4	12.5 ± 21.5	0.978 ± 0.011	1.8 ± 0.8	6.9 ± 5.1
D‐LinkNet	0.963 ± 0.037	3.1 ± 3.2	12.7 ± 14.9	0.978 ± 0.013	1.8 ± 1.2	9.3 ± 10.3
Res‐U‐Net (concat)	0.965 ± 0.030	3.1 ± 2.6	19.8 ± 21.1	0.978 ± 0.013	1.8 ± 1.0	7.2 ± 6.0
Res‐U‐Net (add)	0.964 ± 0.032	3.2 ± 2.7	19.9 ± 21.8	0.978 ± 0.017	1.8 ± 1.2	7.4 ± 6.6
U‐Net	0.920 ± 0.161	5.3 ± 5.1	31.6 ± 32.9	0.978 ± 0.013	1.8 ± 1.0	7.5 ± 5.6
U‐Net (PReLU)	0.961 ± 0.031	3.6 ± 2.7	21.5 ± 21.4	0.979 ± 0.015	1.7 ± 1.1	8.2 ± 9.5
U‐Net (VGG19)	0.709 ± 0.305	12.3 ± 11.1	53.1 ± 47.8	0.975 ± 0.014	2.0 ± 1.1	8.9 ± 7.9

Abbreviations: DSC, Dice similarity coefficient; HD, Hausdorff distance.; MSD, mean surface distance; SD, standard deviation.

**TABLE 3:** Hyperparameters associated with models showing the best performance^a^ in the test dataset.

Model	Image normalization	Learning rate	Kernel size	Depth	Initial filters	Total parameters
DeepLabv3+	z‐score (per image)	0.0001	–	–	–	4.13×10^7^
D‐LinkNet	z‐score (global)	0.0001	–	–	–	4.86×10^7^
Res‐U‐Net (concat)	L_2_	0.0001	3×3	6	16	6.42×10^7^
Res‐U‐Net (add)	L_2_	0.0001	5×5	6	16	7.81×10^7^
U‐Net	z‐score (global)	0.0001	5×5	6	64	3.26×10^8^
U‐Net (PReLU)	z‐score (per image)	0.0001	5×5	6	16	4.06×10^7^
U‐Net (VGG19)	Histogram stretching (global)	0.0001	–	–	–	1.63×10^8^

^a^ Optimal performance was determined by average dice similarity coefficient values.

**FIGURE 3:**
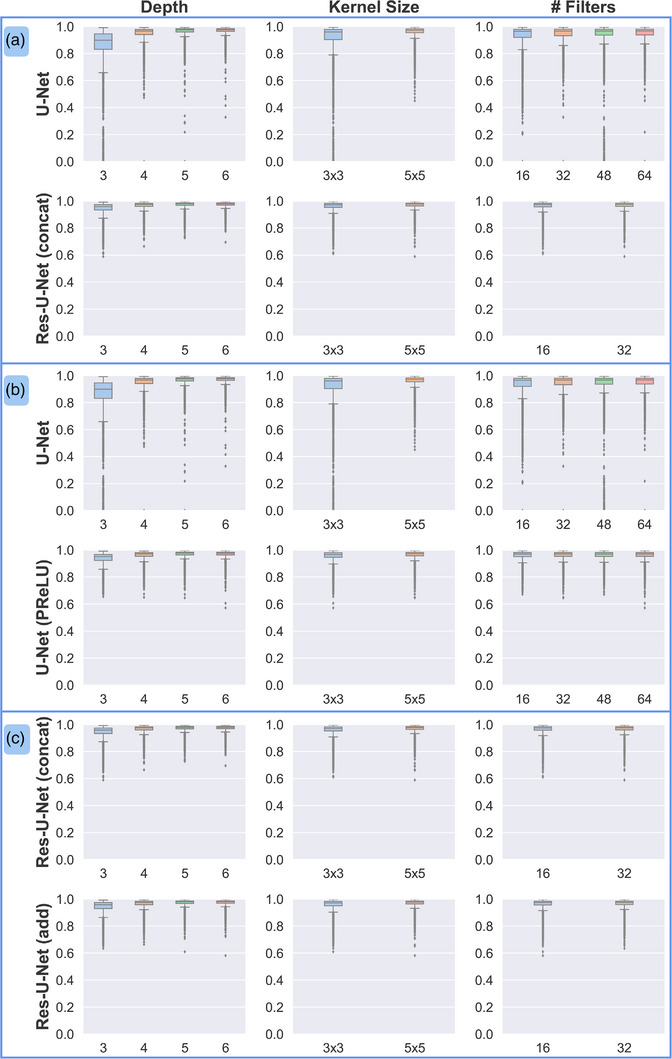
Effect of hyperparameters selected for U‐Net architectures on Dice similarity coefficient (DSC) values in the test set. (a) Comparison of traditional U‐Net and Res‐U‐Net, which employs residual connections. (b) Comparison of ReLU and PReLU activation functions. (c) Comparison of concatenation and element‐wise addition of features in the residual connections of the Res‐U‐Net.

The effects of depth, kernel size, and number of initial filters on DSC values in the test data for the U‐Net–based architectures are shown in Figure 3. Overall, better segmentation accuracy was observed when the depth was increased in the U‐Net architecture (Pearson correlation coefficient 0.38 for DSC, −0.48 for MSD, and −0.52 for HD; Figure 3a). Similarly weak correlations were observed when kernel size was increased from 3×3 to 5×5 (Pearson correlation coefficient 0.16 for DSC, −0.18 for MSD, and −0.19 for HD), although increasing the number of filters used in the first convolutional layer of the U‐Net did not result in a noticeable change in segmentation accuracy (Pearson correlation coefficient −0.05 for DSC, 0.07 for MSD, and 0.05 for HD). For activation functions (Figure 3b), PReLU resulted in statistically superior segmentation agreement to the ground‐truth compared with ReLU (p < 0.0001 for all metrics, paired *t* test; p < 0.05 was considered statistically significant); differences in mean DSC, MSD, and HD values were 0.01, −1.33 mm, and −7.92 mm, respectively. Choosing between concatenating and element‐wise addition of feature maps from residual connections at each convolutional block (Figure 3c) resulted in small mean differences in DSC, MSD, and HD values (−0.001, 0.05 mm, and 0.50 mm, respectively) with minimal improvement using the concatenated networks.

**FIGURE 4:**
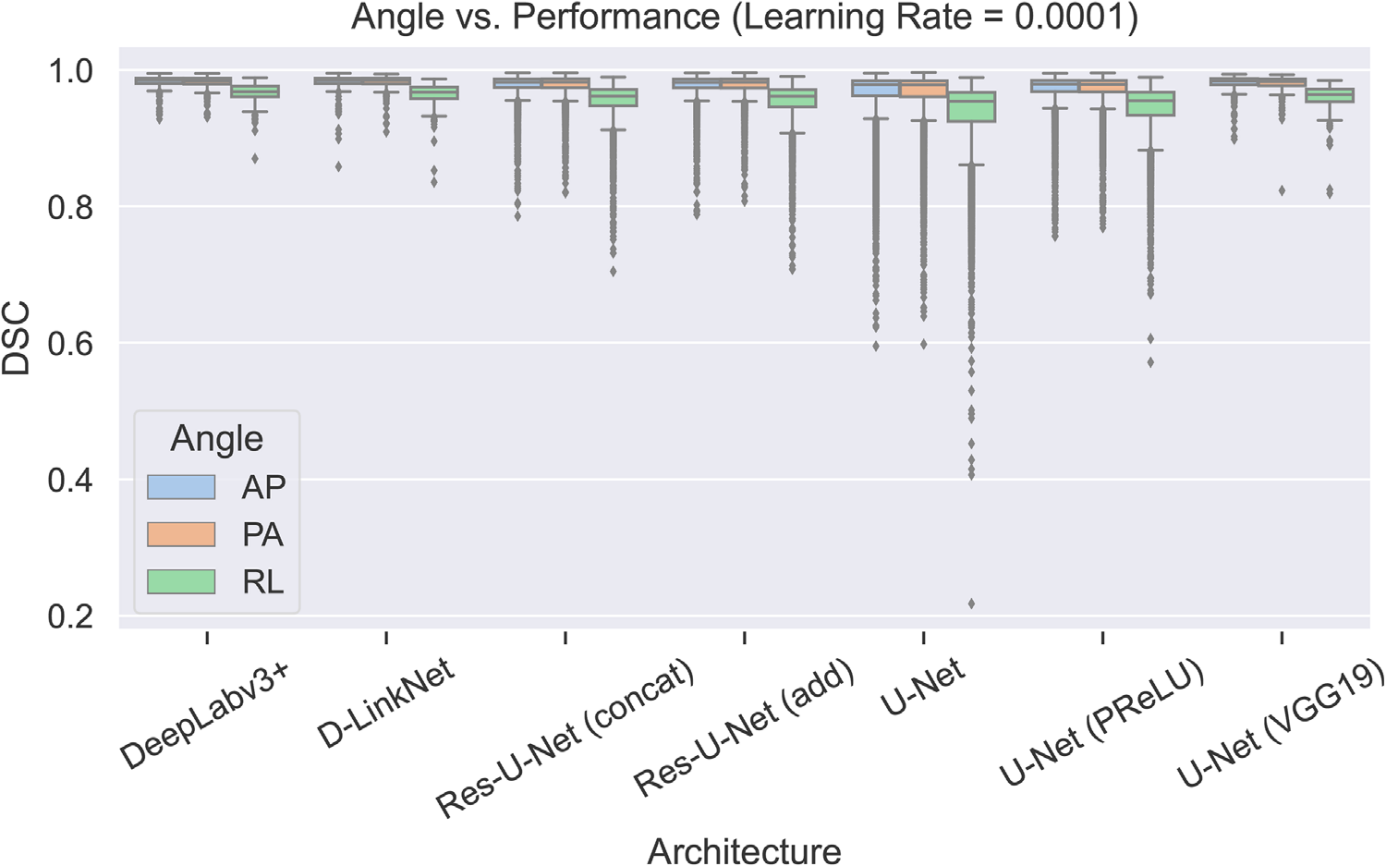
Effects of field angle on Dice similarity coefficient (DSC) values in the test set. For all architectures, median DSC values were lower for right‐lateral (RL) fields than for anterior‐posterior (AP) or posterior‐anterior (PA) fields. For clarity, only results for models trained with a learning rate of 0.0001 are included; the general trend was the same for all models regardless of learning rate.

Beam aperture orientation showed differences in automatically defined fields (Figure 4). The performance of all models was statistically better for AP and PA fields than for lateral (RL) fields (p < 0.0001 for all metrics, paired *t* test). Mean DSC, MSD, and HD values improved by 0.03, −1.1 mm, and −4.7 mm, respectively, for AP beam apertures compared with lateral fields, and by 0.03, −1.3 mm, and −5.7 mm, respectively, for PA beam apertures compared with lateral fields.

## 4 DISCUSSION

In the current study, we performed an exhaustive search of optimal architecture and hyperparameter selection to automatically define cervical cancer radiotherapy treatment beam apertures. Our analysis highlighted some hyperparameter adjustments that were associated with better performance, such as increasing the kernel size, using the PReLU over the ReLU activation function, and increasing network depth for U‐Net‐like architectures. On average, the best performing models resulted in DSC values of 0.98 and MSD values of < 2 mm. These results showed that radiotherapy treatment beam apertures can be automatically defined with high agreement to clinically acceptable beams and could potentially be integrated into fully automated treatment planning workflows.^8^, ^18^

It is worth noting that simply increasing the parameter count is not necessarily enough to improve performance (Table 3). One exception was for U‐Net with the ReLU activation function, where the model with the most parameters (3.26×10^8^) also had the best performance. This is reasonable considering that this architecture has relatively few trainable parameters compared with the Res‐U‐Nets and U‐Net with PReLU architectures. Our results suggest that increasing the number of parameters through larger kernels, repeated convolutions at different receptive fields, or trainable activation functions may generally be more effective than increasing the number of filters to achieve similar parameter counts. Also, two of the best‐performing architectures (DeepLabv3+ and D‐LinkNet), as measured by the average DSC values across all possible model parameter combinations, had the second‐ and third‐fewest parameters; only U‐Net with PReLU activation function achieved its best results at a lower parameter count (Table 2). A high learning rate (0.01) consistently yielded poorer performance than lower learning rates. This is consistent with values observed in the literature, where the learning rate for adaptive optimizers is frequently initialized with a magnitude of 1×10^−3^ or lower, although nonadaptive optimizers such as stochastic gradient descent often do well with a learning rate of 0.01. Furthermore, while a learning rate scheduler was not used in the current study to maintain consistency during training, many studies report improved performance with the use of schedulers.^32^, ^33^, ^34^, ^35^, ^36^

**FIGURE 5:**
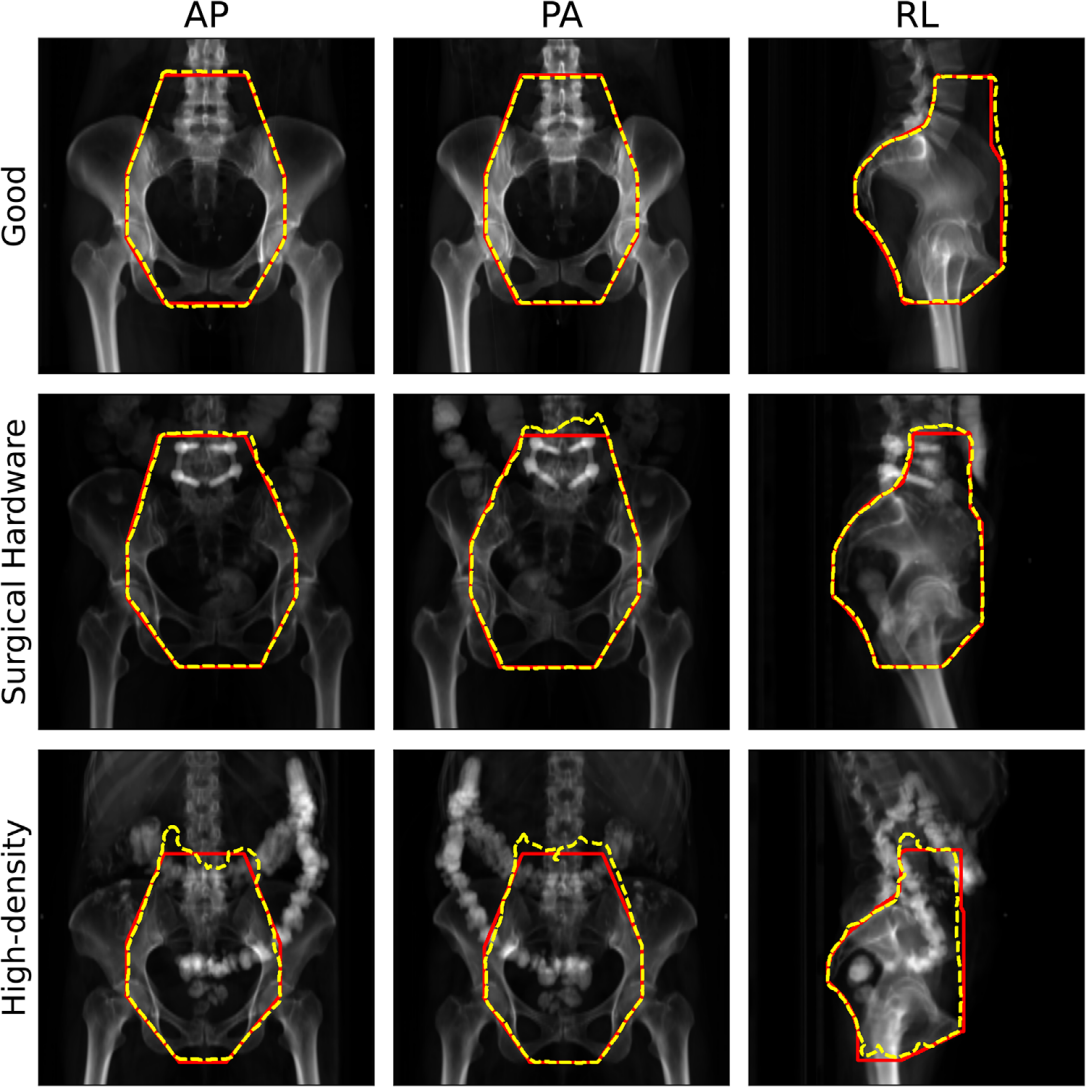
Examples of predictions with “good” and potentially problematic anatomic features in a top‐performing model. All predictions were made using the DeepLabv3+ architecture with optimal hyperparameters identified in Table 3. Red solid borders are physician (ground‐truth) beam apertures, and yellow dashed borders are predicted apertures. AP, anterior‐posterior; PA, posterior‐anterior; RL, right‐lateral field angle.

Patient features can also affect prediction accuracy, especially the presence of surgical hardware or other high‐density materials such as fecal impaction due to the patient's diet (Figure 5). In these cases, the aperture border may appear distorted in or near these high‐intensity regions. It is worth noting that the current analysis intentionally did not use any post‐processing so that models could be directly compared. Simple post‐processing routines would very likely reduce or eliminate the distortions observed with these patient features.

Direct comparison with the literature is challenging owing to differences in anatomic sites or imaging modalities. Using a similar DRR approach, Han et al. described a technique to predict field apertures for whole‐brain radiotherapy^16^ using DeepLabv3+. In a similar 3D CT to 2D planar image projection, Netherton et al. segmented vertebral bodies using X‐Net,^37^ a double‐stacked residual U‐Net with the bottleneck level shared between the residual U‐Nets. Segmentation on 2D x‐ray images is also frequently described in the literature; for instance in lungs,^38^, ^39^, ^40^ various phantom or human anatomic structures,^41^ and recently in COVID‐19 lesions.^42^ However, to the best of our knowledge, no previous study has extensively evaluated selection of architecture or hyperparameters as we have described here; thus, translation across datasets or anatomic sites may be restricted.

The current study has a few limitations. As noted, performance on the lateral fields was slightly lower than on the AP and PA fields. This is likely due to the presence of high‐density materials (e.g., bone, contrast agent, surgical implants), which frequently created image intensity distributions that were less homogenous than those for the AP or PA fields in the DRR projections. The lower foreground (patient anatomy) to background (air) ratio for RL fields may have similarly contributed to the lower performance. Additionally, as previously noted, the choice of optimizer influences the selection of the learning rate. Although Adam performed better with smaller learning rates, it is important to note that this may not generalize to all optimization methods, particularly nonadaptive methods.

GPU memory limitations prevented a subset (784) of U‐Net–like models from training; specifically, Res‐U‐Net with more than 32 filters in the initial convolution. We believe this had little effect on the final result because the best‐performing models used 16 filters in the initial convolution. Similarly, although U‐Net–like architectures performed best when network depth was set to 6 (the deepest evaluated), it is unknown if this trend would have continued because GPU memory did not permit deeper networks to be trained. Because depth implies additional resampling operations and thus allows the network to learn correlating features at additional scales, it is not unreasonable to explore the impact of deeper networks on more capable hardware. However, U‐Net–like architectures often double the number of convolutional filters at each down sampling operation. As previously discussed, increasing parameter count often does not yield better performance; in fact, some studies show comparable performance to state‐of‐the‐art algorithms while significantly restricting parameter count through control of the number of convolutional filters.^43^

In conclusion, the current study, which required over 30,000 computing hours to train 1743 models, is to the best of our knowledge the first to report such an exhaustive search for optimal deep learning architectures and hyperparameters for fully automated beam aperture definition. Of the models evaluated, we identified DeepLabv3+ and D‐LinkNet as the most robust to hyperparameter initialization; however, none of the architectures provided statistically significant improvements when optimal hyperparameters were selected. Among the sets of hyperparameters we identified as providing the best performance, learning rate affected performance for all models. Other optimal hyperparameters varied on a per‐architecture basis, although all U‐Net–like architectures benefited from deeper networks. When using the identified best hyperparameters, our approach is capable of integration into a fully automated treatment planning workflow such as the Radiation Planning Assistant.^18^ Furthermore, all predictions may be validated through a secondary fully automated system for increased confidence.^17^

## AUTHOR CONTRIBUTIONS

Conceptualization, Methodology, Software, Validation, Formal Analysis, Investigation, Resources, Writing—Original Draft, Writing—Review & Editing, Visualization: Skylar Gay. Formal Analysis, Data Curation, Writing—Review & Editing: Kelly D. Kisling. Formal Analysis, Data Curation, Writing—Review & Editing: Brian M. Anderson. Formal Analysis, Data Curation, Writing—Review & Editing: Lifei Zhang. Formal Analysis, Writing—Review & Editing: Dong Joo Rhee. Formal Analysis, Writing—Review & Editing: Callistus Nguyen. Formal Analysis, Writing—Review & Editing: Tucker Netherton. Formal Analysis, Writing—Review & Editing: Jinzhong Yang. Formal Analysis, Data Curation, Writing—Review & Editing: Kristy Brock. Formal Analysis, Data Curation, Writing—Review & Editing: Anuja Jhingran. Formal Analysis, Data Curation, Writing—Review & Editing: Hannah Simonds. Formal Analysis, Data Curation, Writing—Review & Editing: Ann Klopp. Formal Analysis, Data Curation, Writing—Review & Editing: Beth M. Beadle. Conceptualization, Investigation, Writing—Review & Editing, Supervision, Project administration, Funding acquisition: Laurence E. Court. Conceptualization, Investigation, Methodology, Software, Validation, Formal Analysis, Resources, Writing—Original Draft, Writing—Review & Editing, Project administration: Carlos E. Cardenas.

## CONFLICT OF INTEREST STATEMENT

The authors have no conflicts of interest to disclose.
